# 

**Published:** 1884-04

**Authors:** 


					﻿Vol. IV.
APRIL, 1884.
NO. 4.
1PHE
HOMEOPATHIC DHY^ICIAJI,
A MONTHLY JOURNAL OF MEDICAL SCIENCE.
uSeek the Truth: come whence it may, cost what it will."
E. I_.EE, 3VE. E_,
EDITOR.
CONTENTS:
(See inside of Cover.)
PHILADELPHIA:
No. 3109 CHESTNUT STREET.
2ST 2E3 "W O 12, ZEC :
A. L. CHATTERTON, PUBLISHING COMPANY, Publistiek’sJAgents.
LONDON-:
ALFRED HEATH & CO., Sole Agents fob Great Britain,
114 Ebury Street, Eaton Square.
Yearly Subscription, $2.50, invariably in advance.
Entered at the Philadelphia Post-Office as Second-Class Mail Matter.
				

